# Supplementary material for: Genome-wide association studies in a large Korean cohort identify quantitative trait loci for 36 traits and illuminate their genetic architectures
Source: Nat Commun. 2025 May 28;16:4935. doi: 10.1038/s41467-025-59950-5 (PMC12120081; doi:10.1038/s41467-025-59950-5)
Supplement: Supplementary file 1 — Supplementary Information [file 41467_2025_59950_MOESM1_ESM.pdf]

Supplementary Figures

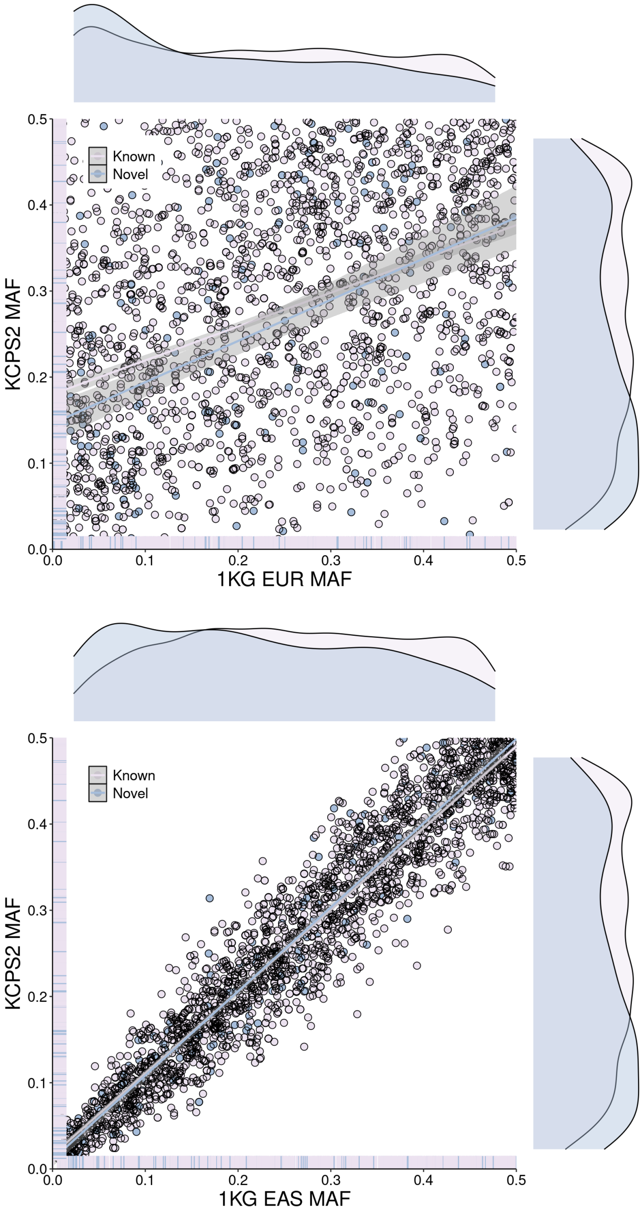

Supplementary Figure 1. MAF comparisons of 2,962 independent genome-wide significant loci in KCPS2 with MAF in 1000 Genomes Project (1KG) European (EUR) and East Asian (EAS) population. The median MAF of the novel loci was 0.118 in 1KG EUR (paired t test P = 2.2e-16) and 0.202 (paired t-test P = 0.0003846) in 1KG EAS.

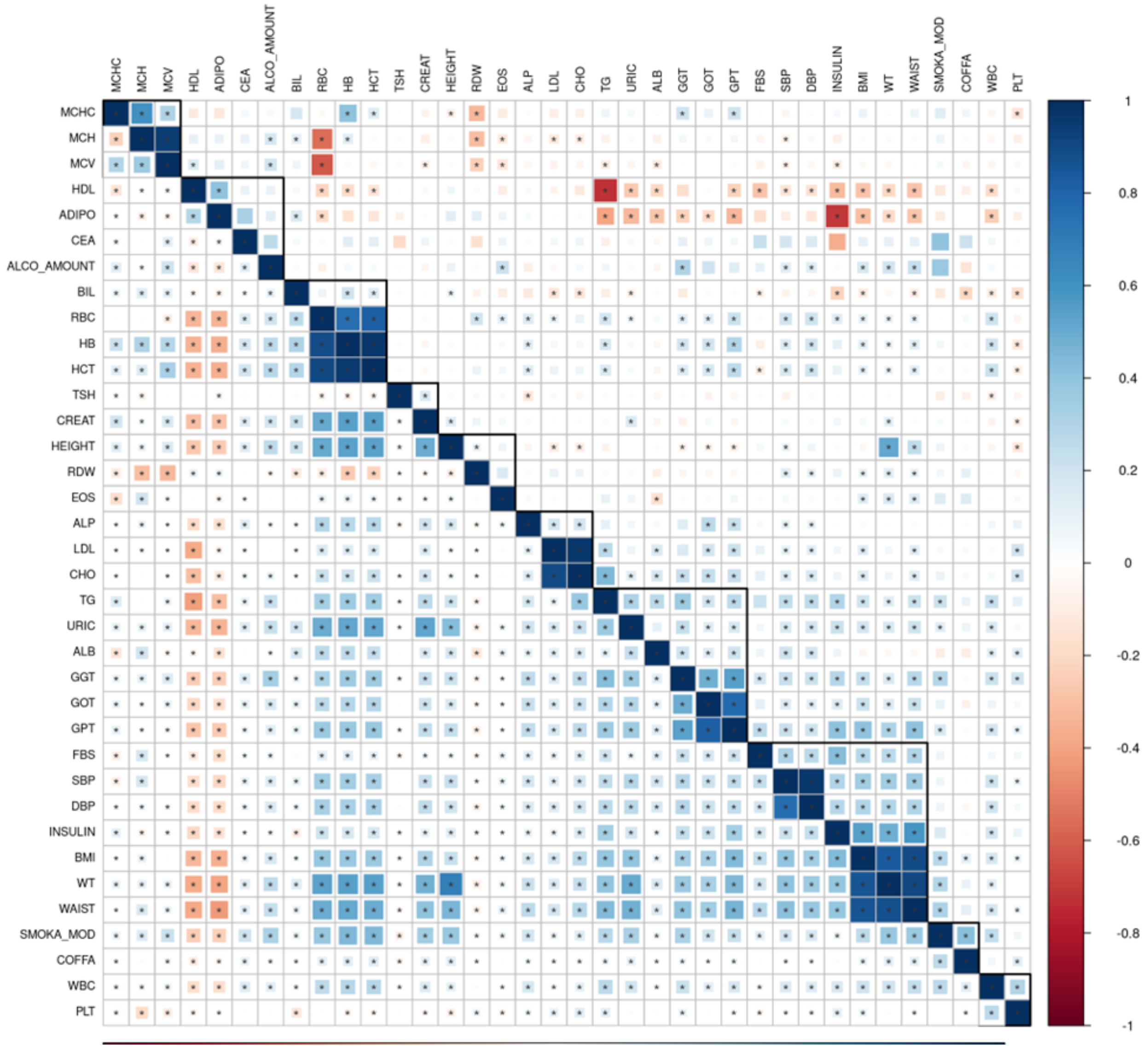

Supplementary Figure 2. Pairwise genetic correlations (r_g, upper diagonal) and phenotypic correlations (r_p, lower diagonal) between the 36 traits in KCPS2. r_g was estimated using bivariate LDSC based on association test statistics from linear regression. Significant r_g and r_p after false discovery rate (FDR<0.05) correction is indicated by an asterisk sign (two-sided Wald test). The complete set of r_g and r_p, is available in Table S5.

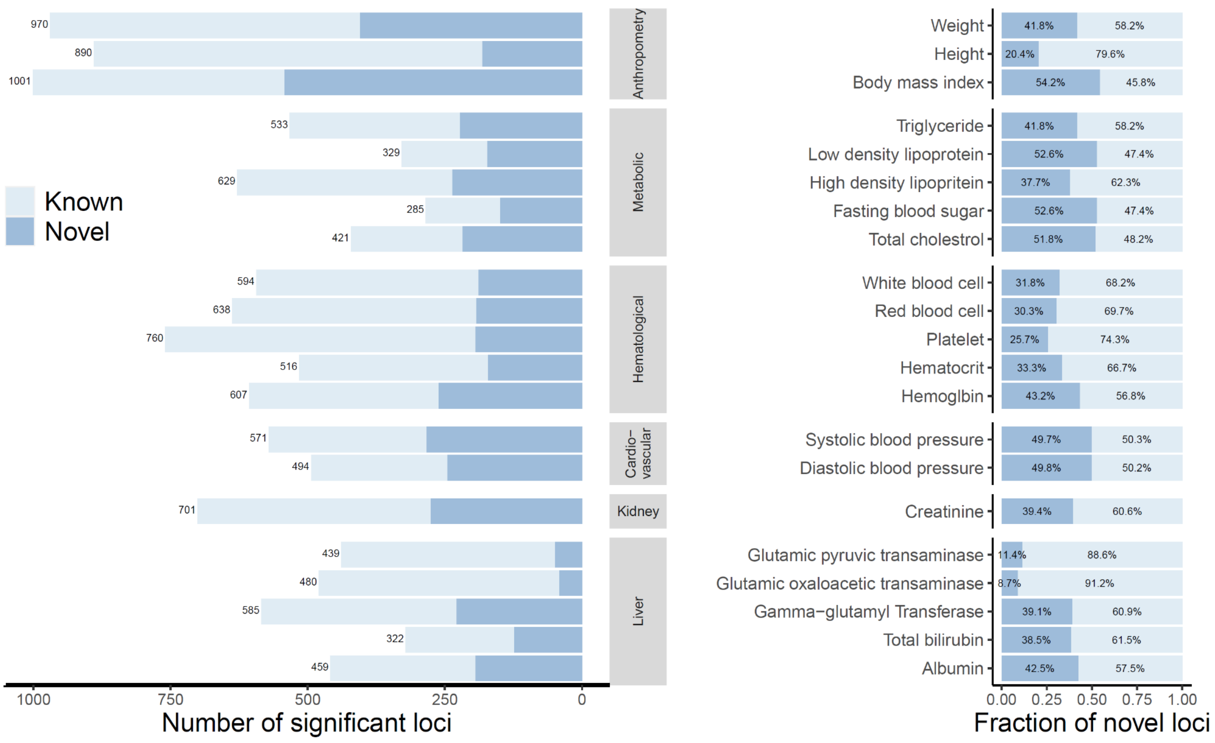

Supplementary Figure 3. Number of known and novel variants identified in the meta-analysis across Korean Cancer Prevention study-II (KCPS2), Biobank Japan (BBJ), Korean Genome and Epidemiology Study (KoGES), Taiwan Biobank (TWB), and UK Biobank (UKB). We identified the association as novel if none of the variants within the locus reached genome-wide significance ($P < 5\times10^{-8}$) in KoGES, BBJ, TWB, or UKB GWAS.

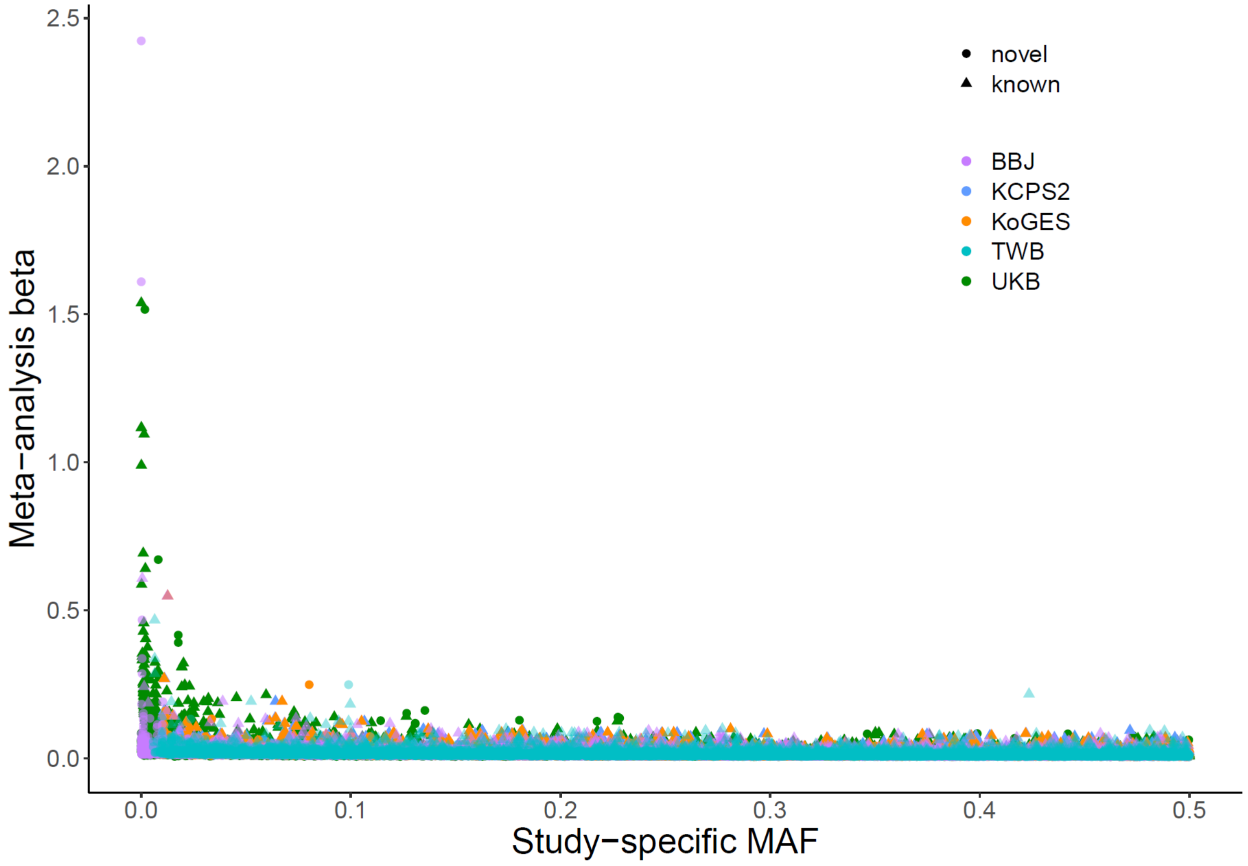

Supplementary Figure 4. Comparisons of study-specific minor allele frequencies and effect sizes estimated from the multi-ancestry meta-analysis. We identified the association as novel if none of the variants within the locus reached genome-wide significance ($P< 5x10^{-8}$) in KoGES, BBJ, TWB, or UKB GWAS.

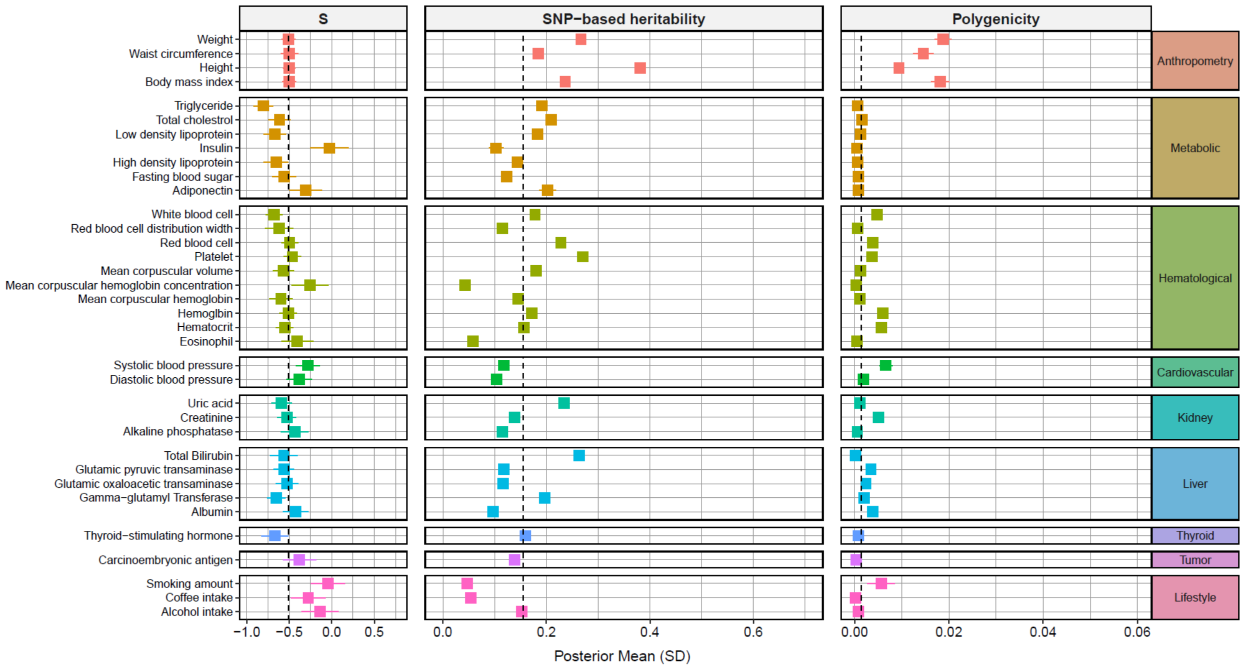

Supplementary Figure 5. Genetic architecture of 36 traits in Korean Cancer Prevention study-II (KCPS2). The dots represent posterior means and horizontal bars represent standard errors of the parameters for each trait. The vertical dashed line shows the median of the estimates across traits.

Supplementary Figure 6. Comparisons of KCPS2 heritability estimates with TWB heritability estimates using different methods and LD reference panel. a) Comparisons between TWB heritability estimates using SbayesS and KCSP2 LD matrix (X-axis) and TWB heritability estimates reported by Chen et al., (2023)[1] using LDSC and in-sample LD from TWB (Y-axis). b) Comparisons between KCPS2 heritability estimates (X-axis) and TWB heritability estimates using SbayesS and KCSP2 LD matrix (Y-axis).

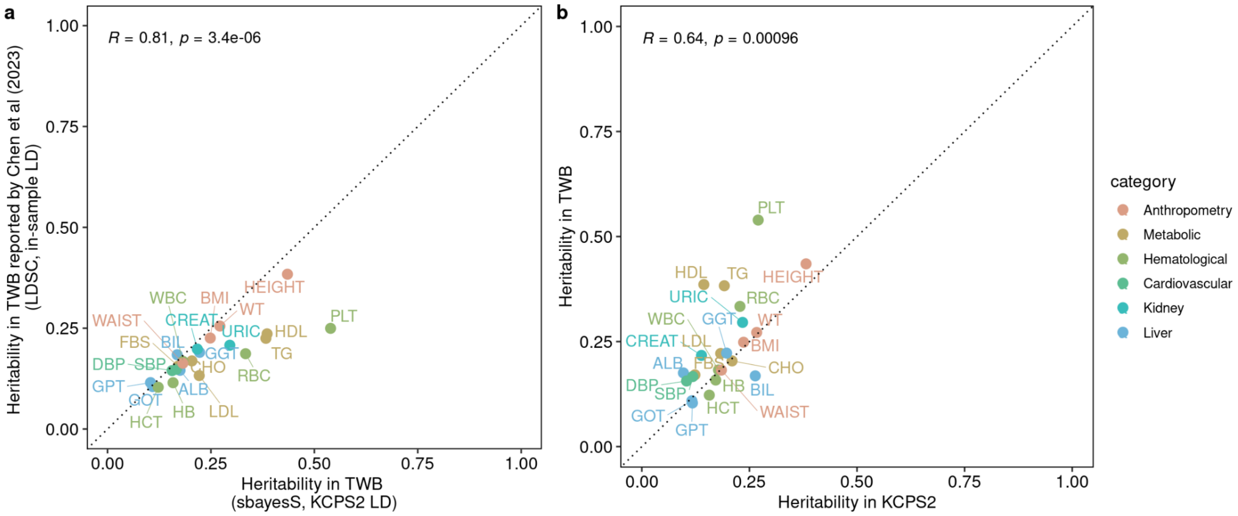

For panel (b), we removed chromosome 2 heritability estimate for total bilirubin in TWB, which led to heritability being greater than 1 (h^2_g=1.12) due to the Mendlian locus in chromosome 2. If we did not remove chromosome 2 heritability estimate, the correlation between heritability in KCPS2 and TWB was 0.56 (*P*=0.0056).

Supplementary Figure 7. Comparison of within- and cross-biobank genetic correlation estimates for 21 quantitative traits in KCPS2, KoGES, BBJ, TWB, and UKBB (r_g). (a-c) The r_g estimates within EAS were computed in LDSC[2] using 1000 Genomes Project EAS reference panel. (d-f) The cross-biobank population genetic effect correlations between KCPS2 and UKB were estimated in Popcorn (v.1.0)[3] using precomputed cross-population scores for EUR and EAS 1000 Genomes Project populations (Tables S9).

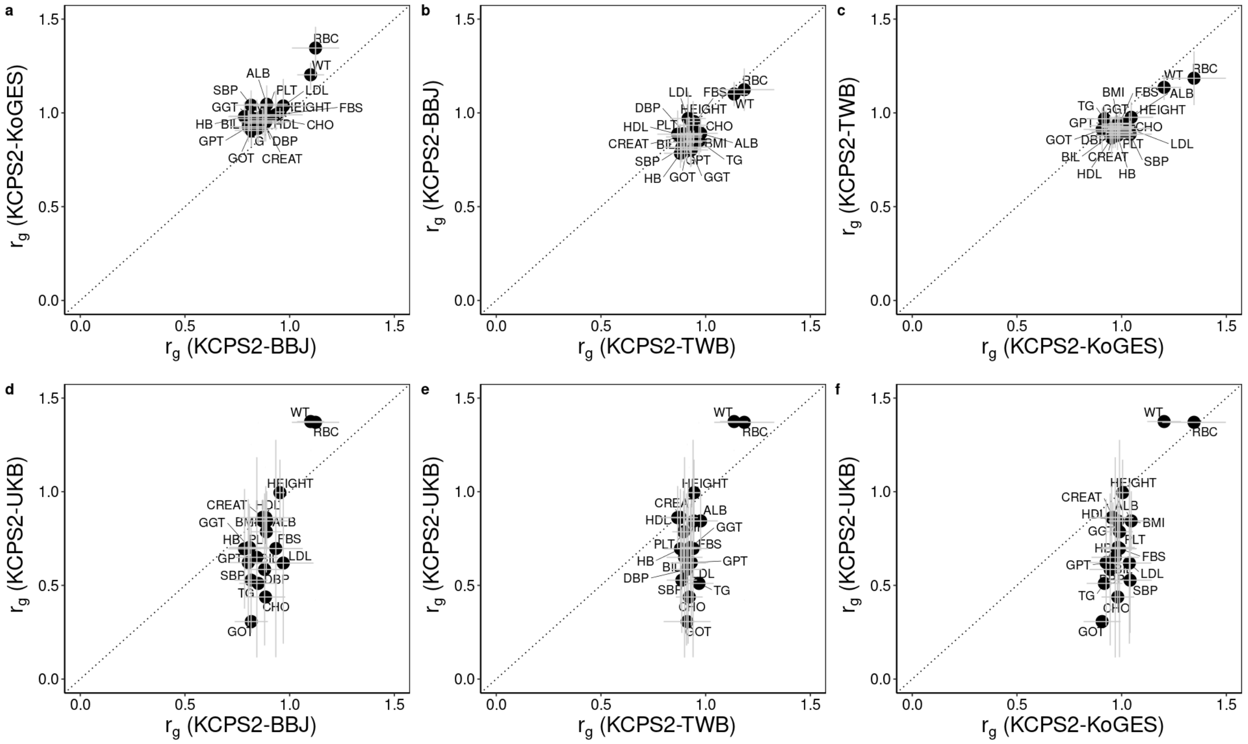

Supplementary Figure 8. Flow chart of identifying novel loci.

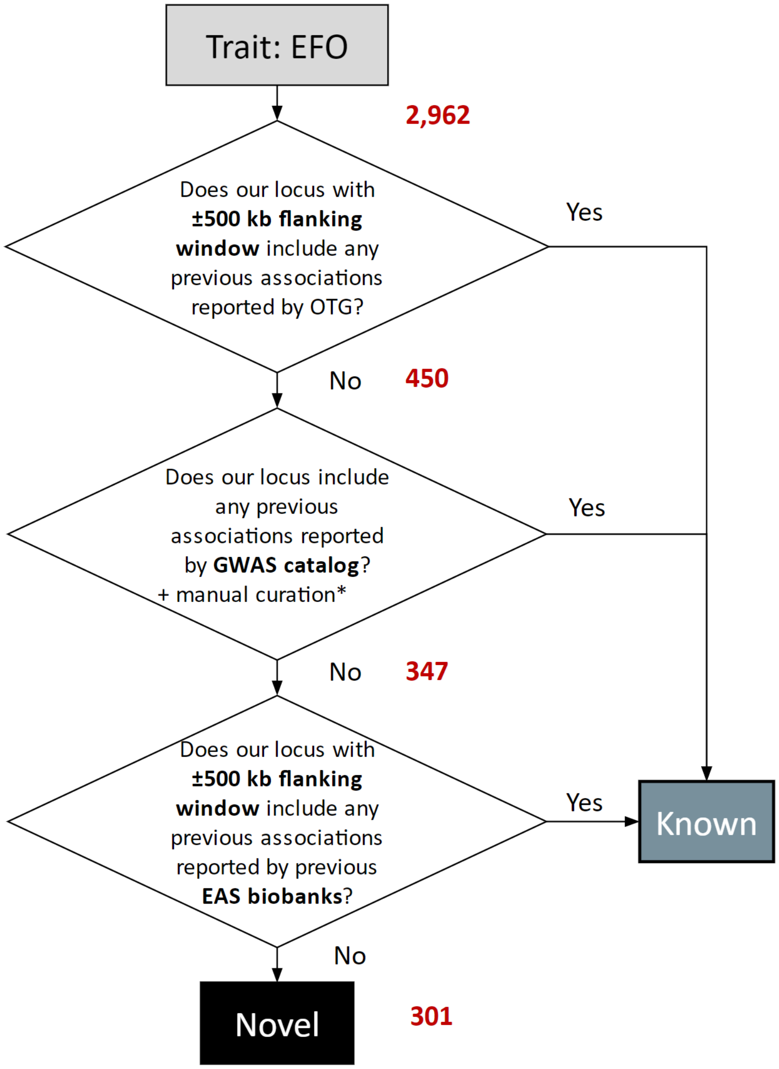

The numbers indicate the number of loci across 36 traits in KCPS2.
*Yengo et al (2022)[4] for height, Sterenborg et al (2024)[5], William et al (2023)[6] for TSH.

References

1. Chen, C.-Y. *et al.* Analysis across Taiwan Biobank, Biobank Japan, and UK Biobank identifies hundreds of novel loci for 36 quantitative traits. *Cell Genom.* **3**, 100436 (2023).
2. ReproGen Consortium *et al.* An atlas of genetic correlations across human diseases and traits. *Nat. Genet.* **47**, 1236–1241 (2015).
3. Brown, B. C., Asian Genetic Epidemiology Network Type 2 Diabetes Consortium, Ye, C. J., Price, A. L. & Zaitlen, N. Transethnic Genetic-Correlation Estimates from Summary Statistics. *Am. J. Hum. Genet.* **99**, 76–88 (2016).
4. Yengo, L. *et al.* A saturated map of common genetic variants associated with human height. *Nature* **610**, 704–712 (2022).
5. Sterenborg, R. B. T. M. *et al.* Multi-trait analysis characterizes the genetics of thyroid function and identifies causal associations with clinical implications. *Nat. Commun.* **15**, 888 (2024).
6. Williams, A. T. *et al.* Genome-wide association study of thyroid-stimulating hormone highlights new genes, pathways and associations with thyroid disease. *Nat. Commun.* **14**, 6713 (2023).
