# Supplementary material for: Genome-wide association studies in a large Korean cohort identify quantitative trait loci for 36 traits and illuminate their genetic architectures
Source: Nat Commun. 2025 May 28;16:4935. doi: 10.1038/s41467-025-59950-5 (PMC12120081; doi:10.1038/s41467-025-59950-5)
Supplement: Supplementary file 2 — Description of Additional Supplementary Files [file 41467_2025_59950_MOESM2_ESM.pdf]

Description of Additional Supplementary Files

Supplementary Data 1: Information on the 36 quantitative traits examined.

Supplementary Data 2: Genome-wide significant loci

Supplementary Data 3: Number of novel KCPS2 GWAS loci using EFO terms

Supplementary Data 4: Information on the pleiotropic genes.

Supplementary Data 5: Pairwise genetic/phenotypic correlation estimates between 36 quantitative traits in KCSP2.

Supplementary Data 6: Meta-analysis results across KCPS2, KoGES, BBJ, TWB, and UKB.

Supplementary Data 7: Number of novel meta-analysis

Supplementary Data 8: Genetic architecture across KCPS2, BBJ, TWB, and UKB.

Supplementary Data 9: Cross-biobank genetic correlations across KCPS2, KoGES, BBJ, TWB, and UKB

Supplementary Data 10: Full results for the fine-mapping and colocalization analysis.

Supplementary Data 11: Sensitivity analysis of fine-mapping analysis using L=5, L=1.

Supplementary Data 12: Number of replicated KCPS2 loci.

Supplementary Data 13: Cohort characteristics.

Supplementary Data 14: Wilcoxon test for genetic architecture comparison
