# Supplementary material for: Genome-wide association studies in a large Korean cohort identify quantitative trait loci for 36 traits and illuminate their genetic architectures
Source: Nat Commun. 2025 May 28;16:4935. doi: 10.1038/s41467-025-59950-5 (PMC12120081; doi:10.1038/s41467-025-59950-5)
Supplement: Supplementary file 4 — Reporting Summary [file 41467_2025_59950_MOESM4_ESM.pdf]

nature portfolio

Corresponding author(s): Peter Kraft

Last updated by author(s): Mar 30, 2025

Reporting Summary

Nature Portfolio wishes to improve the reproducibility of the work that we publish. This form provides structure for consistency and transparency in reporting. For further information on Nature Portfolio policies, see our Editorial Policies and the Editorial Policy Checklist.

Statistics

For all statistical analyses, confirm that the following items are present in the figure legend, table legend, main text, or Methods section.

n/a	Confirmed	
☐	☒	The exact sample size (*n*) for each experimental group/condition, given as a discrete number and unit of measurement
☐	☒	A statement on whether measurements were taken from distinct samples or whether the same sample was measured repeatedly
☐	☒	The statistical test(s) used AND whether they are one- or two-sided<br>*Only common tests should be described solely by name; describe more complex techniques in the Methods section.*
☐	☒	A description of all covariates tested
☐	☒	A description of any assumptions or corrections, such as tests of normality and adjustment for multiple comparisons
☐	☒	A full description of the statistical parameters including central tendency (e.g. means) or other basic estimates (e.g. regression coefficient) AND variation (e.g. standard deviation) or associated estimates of uncertainty (e.g. confidence intervals)
☐	☒	For null hypothesis testing, the test statistic (e.g. *F*, *t*, *r*) with confidence intervals, effect sizes, degrees of freedom and *P* value noted<br>*Give P values as exact values whenever suitable.*
☒	☐	For Bayesian analysis, information on the choice of priors and Markov chain Monte Carlo settings
☐	☒	For hierarchical and complex designs, identification of the appropriate level for tests and full reporting of outcomes
☐	☒	Estimates of effect sizes (e.g. Cohen's *d*, Pearson's *r*), indicating how they were calculated

Our web collection on statistics for biologists contains articles on many of the points above.

Software and code

Policy information about availability of computer code

Data collection	No software was used in data collection.
Data analysis	We used publicly available software for the data analysis: SAIGE(v.1.1.9), FUMA, LDSC (v.1.0.1), R 4.2.3, plink2.0, python3, Hail0.2, METAL (released on 2020-05-05), Popcorn (v.1.0), susieR (0.12.35), IMPUTE5, SHAPEIT4. Analysis code is available at https://doi.org/10.5281/zenodo.15110489.

For manuscripts utilizing custom algorithms or software that are central to the research but not yet described in published literature, software must be made available to editors and reviewers. We strongly encourage code deposition in a community repository (e.g. GitHub). See the Nature Portfolio guidelines for submitting code & software for further information.

Data

Policy information about availability of data

All manuscripts must include a data availability statement. This statement should provide the following information, where applicable:

- Accession codes, unique identifiers, or web links for publicly available datasets
- A description of any restrictions on data availability
- For clinical datasets or third party data, please ensure that the statement adheres to our policy

The individual-level KCPS2 data are not publicly available but potential collaborators are invited to contact the corresponding authors. The GWAS summary statistics generated in this study are publicly available at https://zenodo.org/records/15132424. Data sources for other publicly available GWAS summary statistics are

available in Supplementary Table 13. The summary statistics for KoGES used in this study were downloaded from the KoGES Zenodo (https://zenodo.org/record/7042518), BBJ summary statistics from the Biobank Japan PheWeb (https://pheweb.jp/), TWB summary statistics from GWAS Catalog (https://www.ebi.ac.uk/gwas/publications/38116116), and summary statistics for Europeans in UKB were downloaded from Pan-UK Biobank (https://pan.ukbb.broadinstitute.org/).

Research involving human participants, their data, or biological material

Policy information about studies with human participants or human data. See also policy information about sex, gender (identity/presentation), and sexual orientation and race, ethnicity and racism.

Reporting on sex and gender	Self-reported sex was used as a covariate in our analysis.
Reporting on race, ethnicity, or other socially relevant groupings	For association testing in KCPS2, we adjusted for age, sex, 10 top genetic principal components, and SNP array. For multi-ancestry analyses, we used ancestry-specific GWAS datasets where individuals were grouped by genetic ancestry.
Population characteristics	The Korean Cancer Prevention Study-II Biobank (KCPS2) is a prospective cohort study based in Korea with genotype data and measurements of a wide range of phenotypes collected from 153,950 subjects. Mean age of participants at recruitment was 41.7 yr old, and 40% were female. All participants in the KCPS2 were genotyped using either the Illumina Global Screening Array (GSA) v2.0 (78,260 samples) or the Korean Chip array v1 (90,245 samples). The description of the dataset for GWAS from other biobanks can be found in the original publications as listed in Supplementary Table 14.
Recruitment	Participants in KCPS2 undertook routine health assessments at nationwide health promotion centers between 2004 and 2013. Approximately 90% of participants were recruited from the Seoul and Gyeonggi regions, where about 40% of the South Korean population resides (around 19 million people). Therefore, the cohort may not provide generalizable estimates for the entire population, particularly for characteristics that vary geographically across the country. We also note that each biobank used in this manuscript has specific population context: KCPS2, KoGES, TWB, and UKB are population-based cohorts, while BBJ is a hospital-based cohort. The coherent results across five biobanks mitigated concerns over potential biases.
Ethics oversight	All KCPS2 participants provided written informed consent approved from ethics committees of Yonsei University.

Note that full information on the approval of the study protocol must also be provided in the manuscript.

Field-specific reporting

Please select the one below that is the best fit for your research. If you are not sure, read the appropriate sections before making your selection.

☒ Life sciences ☐ Behavioural & social sciences ☐ Ecological, evolutionary & environmental sciences

For a reference copy of the document with all sections, see nature.com/documents/nr-reporting-summary-flat.pdf

Life sciences study design

All studies must disclose on these points even when the disclosure is negative.

Sample size	The sample sizes used in the GWASs for this study are summarized in Supplementary Tables 1 and 6. While we did not perform a formal sample size calculation, we included the maximum number of individuals from each cohort who met the quality control (QC) criteria. This approach ensured that statistical power was maximized for each cohort, and we further enhanced power through cross-population meta-analysis.
Data exclusions	Samples were selected based on quality control criteria specific to each cohort, as detailed in the Methods section.
Replication	We validated the signals identified in the KCPS2 GWASs by comparing them with corresponding but independent GWASs conducted in KoGES, BBJ, TWB, and the UK Biobank. The results demonstrated high replicability as described in Supplementary Table 12.
Randomization	Randomization was not applicable in this study, as it is a genotype-phenotype association study. All available samples with genotype and phenotype data were included in the analysis.
Blinding	Blinding was not employed because the study involved no interventions and focused solely on genotype-phenotype associations.

Reporting for specific materials, systems and methods

We require information from authors about some types of materials, experimental systems and methods used in many studies. Here, indicate whether each material, system or method listed is relevant to your study. If you are not sure if a list item applies to your research, read the appropriate section before selecting a response.

Materials & experimental systems

n/a	Involved in the study
☒	☐ Antibodies
☒	☐ Eukaryotic cell lines
☒	☐ Palaeontology and archaeology
☒	☐ Animals and other organisms
☒	☐ Clinical data
☒	☐ Dual use research of concern
☒	☐ Plants

Methods

n/a	Involved in the study
☒	☐ ChIP-seq
☒	☐ Flow cytometry
☒	☐ MRI-based neuroimaging

Plants

Seed stocks

Report on the source of all seed stocks or other plant material used. If applicable, state the seed stock centre and catalogue number. If plant specimens were collected from the field, describe the collection location, date and sampling procedures.

Novel plant genotypes

Describe the methods by which all novel plant genotypes were produced. This includes those generated by transgenic approaches, gene editing, chemical/radiation-based mutagenesis and hybridization. For transgenic lines, describe the transformation method, the number of independent lines analyzed and the generation upon which experiments were performed. For gene-edited lines, describe the editor used, the endogenous sequence targeted for editing, the targeting guide RNA sequence (if applicable) and how the editor was applied.

Authentication

Describe any authentication procedures for each seed stock used or novel genotype generated. Describe any experiments used to assess the effect of a mutation and, where applicable, how potential secondary effects (e.g. second site T-DNA insertions, mosiacism, off-target gene editing) were examined.
